# USING AGILE METHODOLOGY AND NUDGE STRATEGIES TO IMPROVE ENROLLMENT IN CLINICAL TRIALS

**DOI:** 10.1093/geroni/igac059.2409

**Published:** 2022-12-20

**Authors:** Peggy Bylund, Jade Mehta, Nandini Mathavan, Kimberly Trowbridge, Britain Taylor, Husam El Sharu, Noll Campbell

**Affiliations:** Regenstrief Institute, Inc., Indianapolis, Indiana, United States; Indiana University School of Medicine, Indianapolis, Indiana, United States; Indiana University School of Medicine/Center for Health Innovation and Implementation Science, Indianapolis, Indiana, United States; Regenstrief Institute, Inc., Indianapolis, Indiana, United States; Indiana University Bloomington, Bloomington, Indiana, United States; Indiana University, Indianapoilis, Indiana, United States; Purdue University, Indianapolis, Indiana, United States

## Abstract

The enrollment of human subjects is crucial for the success of clinical trials. In the ongoing “Reducing the Risk of Dementia through Deprescribing” trial, the initial approach for enrolling subjects did not meet expected goals in the first 6 months, creating the need for innovative nudge strategies. We used an Agile methodology as the framework to understand the problem, then find and implement a solution. Our study aimed to examine the effectiveness of utilizing a texting nudge to enhance post-agreement recruitment of subjects with cognitive impairments. Prior to enrollment, eligible potential participants were contacted using a texting nudge. Potential participants received a second contact call to remind subjects of the enrollment appointment, introduce the person and the phone number that would call them, and the option of confirming or rescheduling. During the 1-week text-message experiment, 8 out of 9 subjects who agreed to participate in the study and received the text message enrolled, yielding an 89% post-agreement enrollment rate compared to a baseline rate of 44% prior to introducing this nudge. After implementing into the standard operating procedures, the 6-month average rate of enrollment among those that agreed rose to 80%, nearly doubling the rate from the first 6 months of the study and quadrupling the number enrolled each month. Inadequate recruitment has necessitated the use of innovative recruitment methods. Using the Agile problem-solving mindset, the texting nudge was developed to leverage the behavioral influences of the messenger, social commitments, priming and affect to increase subject enrollment.

